# Description of an Improved Instrument for the Fistula in Ano

**Published:** 1790

**Authors:** 


### II. *Description of an improved Instrument for the Fistula in Ano. Communicated in a Letter to Dr.* Simmons, *F. R. S. by Mr.* J. Savigny, *Surgical-Instrument Maker in London.*

IT has for a long time been a subject of complaint that the knife usually employed, by surgeons, in the operation for the fistula in ano, though very ingeniously contrived, and in most respects adequate to its intended purpose, is materially defective in one important particular, namely, the difficulty the operator experiences in forcing the button, at its extremity, through a diseased gut; a circumstance which must necessarily render the operation much more painful than it would otherwise be to the patient.

This complaint has induced me to direct my attention to the subject; and I flatter myself that the instrument I now beg leave, Sir, to submit to your consideration (and which has been honoured with the approbation of many eminent surgeons who have employed it in their practice) will be found to remedy the above-mentioned inconvenience.

It

It is compofed of two blades (one fixed, the other moveable; the former with a cutting edge and blunt point, and the latter with an exquifitely fharp point, and blunt in all its other dimenfions) moft accurately adjufted to each other, and retained in a parallel pofition by a fcrew and fliding fpring, which the annexed figures and the following references to them will more fully explain:

FIGURE 1. Reprefents the inftrument in its proper ftate to be introduced into the finus, with the fliding blade fo far advanced that its fharp point is exactly on a level with and protected by the blunt one of the cutting blade.

FIG. 2. Gives it in another point of view, when it is fuppofed to be introduced as far as neceffary, with the fharp point protruded beyond the blunt one, to facilitate the paffage of the button through the gut.

FIG. 3. Exhibits the fliding blade withdrawn, after having conducted the button through its deftination, with the edge of the cutting blade expofed, and free from all impediment, to finifh the operation.

*Pall Mall,*
July 2, 1790.

III. *An*

